# Is marijuana use associated with decreased use of prescription opioids? Toxicological findings from two US national samples of drivers

**DOI:** 10.1186/s13011-020-00257-7

**Published:** 2020-02-17

**Authors:** Guohua Li, Stanford Chihuri

**Affiliations:** 1 Department of Anesthesiology, Columbia University Vagelos College of Physicians and Surgeons, 622 West 168th St, PH5-505, New York, NY 10032 USA; 2 Department of Epidemiology, Columbia University Mailman School of Public Health, New York, NY USA

**Keywords:** Alcohol, Harm reduction, Marijuana, Prescription opioids, Substitution

## Abstract

**Background:**

State governments in the United States are increasingly viewing marijuana legalization as a policy option for controlling the opioid epidemic under the premise that marijuana is a less harmful substitute for opioids. The purpose of this study is to assess whether marijuana use is associated with decreased odds of prescription opioid use.

**Methods:**

A cross-sectional study design was applied to toxicological testing data from two national samples of drivers: 1) the 2011–2016 Fatality Analysis Reporting System (FARS) and 2) the 2013–2014 National Roadside Survey of Alcohol and Drug Use by Drivers (NRS). Adjusted odds ratios (ORs) and 95% confidence intervals (CIs) estimated from multivariable logistic regression models were used to assess the associations of marijuana use with prescription opioid use and alcohol use.

**Results:**

Among the 47,602 drivers from the FARS, 15.7% tested positive for marijuana and 6.9% positive for prescription opioids. Compared with drivers testing negative for marijuana, those testing positive for marijuana were 28% more likely to test positive for prescription opioids (adjusted OR = 1.28, 95% CI = 1.15–1.42). Among the 7881 drivers from the NRS, 7.9% tested positive for marijuana and 4.5% positive for prescription opioids. Relative to drivers testing negative for marijuana, those testing positive for marijuana were twice as likely to test positive for prescription opioids (adjusted OR = 2.03, 95% CI = 1.29–3.20). In both study samples, marijuana use was associated with significantly increased odds of alcohol positivity.

**Conclusions:**

Drivers who test positive for marijuana are significantly more likely to test positive for prescription opioids. Longitudinal studies with rigorous designs and toxicological testing data are needed to further address the substitution hypothesis between marijuana and prescription opioids.

## Background

The United States is grappling with an ongoing opioid epidemic that started more than 20 years ago. The epidemic was fueled initially by prescription opioids, followed by heroin and then by fentanyl and analogs. Now, the epidemic is entering another phase marked by the resurgence of polydrug use with stimulants such as cocaine and methamphetamines in addition to opioids [1–5]. In 2017, US healthcare providers wrote 191 million opioid prescriptions; a rate of 58.7 prescriptions per 100 persons [6]. Although the number of all opioid-related deaths dropped by 2% between 2017 and 2018 [2, 7], 130 people die every day from opioid overdose [8]; of these, 46 die from prescription opioid overdose and more than 1000 people are treated for prescription opioid misuse in emergency departments across the United States daily [7, 9, 10]. The Centers for Disease Control and Prevention estimates that the annual total economic burden of the opioid epidemic, including lost productivity, costs of addiction treatment, criminal justice proceedings, and direct and indirect healthcare costs, is $78.5 billion [11].

In response to the opioid epidemic, federal, state and local governments in the United States have been implementing a wide array of strategies such as improving access to treatment for opioid use disorders, expanding distribution of naloxone, and enhancing prescription drug monitoring programs [1, 3, 4, 9, 10]. Another emerging harm-reduction strategy is to substitute less addictive non-opioid alternatives for prescription opioids [12]. In general, substitution of psychoactive substances is a well-known harm-reduction strategy, such as using nicotine patches in lieu of cigarettes or using methadone for treating heroin addiction [13, 14]. Under the same premise, marijuana is suggested as a less harmful substitute for prescription opioids in the treatment of pain [15, 16]. Recent regulations passed in New York and Illinois allow authorized patients with an opioid prescription to legally purchase medical marijuana at a registered dispensary [12, 17]. As of July 2019, 34 states and the District of Columbia have legalized medical marijuana for patients with certain diseases and 10 states and the District of Columbia have legalized marijuana for recreational use among adults [18], with more states moving toward legalizing recreational marijuana. One reason commonly cited by proponents of marijuana legalization is that the policy may help control the opioid epidemic, assuming that marijuana and prescription opioids are substitutive substances, i.e., increased use of marijuana would lead to decreased use of prescription opioids [19, 20].

Studies assessing the substitution hypothesis have generated conflicting results and debate [21–24]. For instance, several small surveys of patients with chronic pain have reported high rates of marijuana substitution for prescription opioids [19, 25–32], while a 4-year cohort study [33] and a national survey [34] found no evidence of substitution. Conversely, other studies have found state medical marijuana laws to be associated with reductions in opioid prescriptions filled [35, 36], opioids-related hospitalizations [37] and mortality [38, 39]. A recent meta-analysis concluded that implementation of medical marijuana laws was associated with a modest 7% reduction in opioid prescriptions dispensed, indicating that marijuana is unlikely a major substitute for prescription opioids [40]. However, aggregate data on marijuana laws and self-reported data on drug use are susceptible to biases and are inadequate for understanding individual-level drug substitution behaviors [41]. The purpose of this study was to assess the association of marijuana use with the odds of prescription opioid use based on toxicological testing data from two US national samples of drivers.

## Methods

### Data sources

Data for this study came from two sources: the Fatality Analysis Reporting System (FARS) and the National Roadside Survey of Drug Use by Drivers (NRS). Both the FARS and the NRS are administered by the National Highway Traffic Safety Administration of the US Department of Transportation. The FARS records all motor vehicle crashes that occur on US public roadways that result in at least one fatality (i.e., driver, passenger or pedestrian) within 30 days in all 50 states, the District of Columbia, and Puerto Rico [42]. The FARS contains more than 140 data elements for each fatal crash, obtained from police crash investigation reports, coroner/medical examiner reports, state driver licensing records, emergency medical service reports, and death certificates [42, 43]. Data elements include variables indicating crash characteristics and environmental conditions at the time of crash (e.g., manner of collision, roadway type, road and weather conditions, time, and date of crash), description of all persons involved (e.g., age, sex, restraint use, and injury severity), and vehicle characteristics (e.g., vehicle type, weight rating, make, model and year) [44]. In addition, the FARS collects toxicological testing data for drivers and others involved in the crash. FARS analysts enter the data into electronic files that are automatically checked for consistency and acceptable ranges [42]. Further, several programs continually monitor the data and perform quality checks to improve the accuracy and completeness [42].

Incepted in 1973 and conducted approximately every 7 years, the NRS was initially designed to estimate the prevalence of alcohol use among non-commercial drivers. Since 2007, the NRS has been expanded to test for both alcohol and drugs [44]. Drivers are selected randomly based on a multistage sampling method that includes four levels in a descending hierarchy: primary sampling units, police jurisdictions, survey locations and passing-by drivers [45]. The most recent NRS was conducted during June 7, 2013 through March 30, 2014 at 300 locations in contiguous states across 4 US regions (Northeast, Midwest, West, and South). At the selected survey locations, verbally consented drivers were administered standardized questionnaires to collect data about their demographic characteristics, drinking behavior, drug use, annual mileage, origin and destination of trip, and asked to provide a sample of oral fluid for drug testing as well as a breath sample for alcohol testing [44, 45]. The 2013–2014 NRS was conducted during a 2-h Friday daytime session (either 9:30 am to 11:30 am or 1:30 pm to 3:30 pm) at 60 locations and during four 2-h nighttime periods (10 pm to midnight and 1 am to 3 am on Friday and Saturday nights) at 240 locations, with a response rate of 79.3% [45]. The study design and methods for the 2013–2014 NRS are described in detail elsewhere [45].

### Drug and alcohol testing

In FARS, toxicological testing was conducted by the respective medico-legal office such as the medical examiner or coroner [46, 47]. Currently, 21 states and the District of Columbia have medical examiner systems, 9 have coroner systems, and 20 have mixed medical examiner and coroner systems responsible for certifying deaths [47, 48]. The FARS database records up to three nonalcohol drugs per driver [42, 49]. If a driver tests positive for multiple drugs, FARS records the detected drugs in the following priority order: narcotics, depressants, stimulants, marijuana, and other [42, 46]. Prescription opioids are classified as Schedule II substances and are recorded under the narcotics category in FARS [50]. In this study, prescription opioids from FARS refer to oral or injectable formulations of codeine, diphenoxylate, fentanyl, hydrocodone, hydromorphone, meperidine, methadone, morphine, oxycodone, oxymorphone, and propoxyphene. In FARS, marijuana refers to cannabinoids such as delta-9-tetrahydrocannabinol (THC), hashish, hashish oil, delta 9 or marinol [43]. If a metabolite was detected, only the parent drug was recorded. Drug tests were performed on blood specimens through liquid/gas chromatography, mass spectrometry, and radioimmunoassay techniques [46, 49]. Blood alcohol concentrations (BACs) were also assessed using liquid/gas chromatography and mass spectrometry techniques where BAC ≥0.01 g/dL was defined as positive [42].

In the 2013–2014 NRS, toxicological testing was performed on oral fluid samples. The oral fluid samples were first screened using enzyme-linked immunosorbent assay (ELISA) micro-plate technology and then confirmed using liquid/gas chromatography and mass spectrometry techniques [45]. The oral fluid test used in the NRS is a highly valid method for detecting the presence of THC in the blood [51]. Marijuana refers to cannabinoids such as THC, THC-COOH, or synthetic marijuana. The minimum detectable screening concentration for marijuana using the ELISA test was 4 ng/ml. Samples with positive marijuana screening results (≥ 4 ng/ml) were further subjected to a more sensitive and more specific confirmation test with a minimum detectable concentration of 2 ng/ml. If the confirmation test was positive (≥2 ng/ml), the sample was regarded as positive, otherwise it was regarded as negative. Complete laboratory procedures for determining marijuana positivity in oral fluid samples are described elsewhere [52]. In the 2013–2014 NRS, there were 22 types of oral or injectable formulations of prescription opioids tested such as codeine, hydrocodone, meperidine, morphine, and oxycodone [45]. The minimum detectable screening concentration for prescription opioids varied by type and ranged from 1 to 50 ng/ml, while minimum detectable confirmation concentration ranged from 0.5 to 25 ng/ml [45]. Complete laboratory procedures for determining opioid positivity in oral fluid samples are described elsewhere [53]. Blood alcohol concentrations (BACs) were assessed using the evidential breath test device where BAC ≥0.02 g/dL was defined as positive [45].

### Study design and analysis

We used a cross-sectional study design to assess whether use of marijuana was associated with decreased odds of prescription opioid use when controlling for alcohol use and demographic characteristics. Data from the 2011–2016 FARS and the 2013–2014 NRS were analyzed separately. The 2011–2016 FARS study sample consisted of 47,602 fatally injured drivers aged 15 years and older who died at the crash scene, exclusive of 149,537 drivers who survived the crashes, 53,574 fatally injured drivers who died on arrival at hospitals or after, 265 fatally injured drivers who were younger than 15 years, and 28,210 fatally injured drivers with missing toxicological testing data. We included only drivers who died at the crash scene to avoid potential biases from post-crash drug metabolism and opioid-based medications administered by healthcare providers. The 2013–2014 NRS study sample comprised 7881 drivers aged 16 years and older who provided oral fluid samples for drug testing.

Analyses proceeded from univariate and bivariate analysis to multivariable logistic regression modeling. Frequency distribution of marijuana, prescription opioid, and alcohol use as indicated by toxicological testing results, and driver characteristics (age, sex, and region) were computed. Estimated crude and adjusted odds ratios (ORs) with corresponding 95% confidence intervals (95% CIs) were used to assess the association of marijuana use with prescription opioid use and alcohol use. Further, stratified analyses were conducted to evaluate the association of marijuana use with prescription opioid use and alcohol use across strata of driver characteristics.

For analyses of the NRS data, survey procedures accounting for clustering within each primary sampling unit were used to obtain valid variance estimates. Specifically, we used the Taylor series (linearization) method in the SURVEYLOGISTIC procedure to estimate the covariance matrix of the regression coefficients. Analytic survey weights based on stratified sampling were used to generalize estimates for the US driver population. All analyses were performed using SAS, version 9.4 software (SAS Institute, Inc., Cary, North Carolina).

## Results

Overall, 47,602 drivers from FARS and 7881 drivers from NRS were included in the study. Compared to drivers excluded due to lack of drug testing data in the FARS, those included were more likely to be from the Southern region (27.5% vs. 22.0%, *p* < 0.001), and more likely to be younger (mean = 41.0 ±17.4 vs. 42.7 ±18.2 yr, *p* < 0.001), but did not differ significantly in the distributions of sex and blood alcohol concentrations. Compared to excluded drivers in the NRS, those included were more likely to be from the Northeast region, (26.1% vs 22.6%, *p* < 0.001), and less likely to be male (58.4 vs. 62.4%, *p* < 0.001), but did not differ significantly in the distributions of age and blood alcohol concentrations.

Among the 47,602 drivers in the FARS sample, 6.9% tested positive for prescription opioids, 15.7% positive for marijuana, and 42.0% positive for alcohol. The majority of the drivers were male, non-Hispanic white, and under 40 years of age (Table 1). Overall, 16.1% of the 3278 opioid-positive drivers and 14.6% of the 44,324 opioid-negative drivers tested positive for marijuana, yielding a crude OR of 1.03 (95% CI = 0.94–1.14). Drivers who tested positive for alcohol were less likely to test positive for prescription opioids (crude OR = 0.69, 95% CI = 0.64–0.74). Female, non-Hispanic white drivers aged 25 years and older had significantly elevated risk of testing positive for prescription opioids (Table 2). Compared with drivers testing negative for marijuana, those testing positive for marijuana were 28% more likely to test positive for prescription opioids (adjusted OR = 1.28, 95% CI = 1.15–1.42) (Table 2) and 50% more likely to test positive for alcohol (adjusted OR = 1.50, 95% CI = 1.42–1.58).

**Table 1:** Characteristics of the study samples, 2011–2016 Fatality Analysis Reporting System (FARS) and 2013–2014 National Roadside Survey of Alcohol and Drug Use by Drivers (NRS)

Characteristic	FARS^a^ (*n* = 47,602) %	NRS^b^ (*n* = 7881) %^c^
Age (years)		
15–24	21.6	28.1
25–39	30.5	33.4
40–64	37.0	33.2
≥ 65	10.9	5.3
Sex		
Male	77.7	56.6
Female	22.3	43.4
Race/ethnicity		
Non-Hispanic White	83.8	61.0
Non-Hispanic Black	11.5	25.7
Other	4.7	13.2
Geographic region		
South	43.8	41.1
Midwest	21.8	20.5
Northeast	11.3	17.5
West	23.1	20.9
Testing positive for prescription opioids		
No	93.1	95.5
Yes	6.9	4.5
Testing positive for marijuana		
No	84.3	92.1
Yes	15.7	7.9
Testing positive for alcohol		
No	58.0	94.7
Yes	42.0	5.3

^a^There were 7 drivers with missing data on gender and 4616 on race from the FARS

^b^There were 204 drivers with missing data on age, 106 on gender, and 89 on race from the NRS

^c^Percentage of drivers weighted for the US driver population

**Table 2:** Estimated crude and adjusted odds ratios and 95% confidence intervals of prescription opioid positivity according to driver characteristics, marijuana positivity and alcohol positivity, 2011–16 Fatality Analysis Reporting System (FARS) and 2013–14 National Roadside Survey of Alcohol and Drug Use by Drivers (NRS)

Characteristic	FARS^a^				NRS^b^			
	Prescription Opioids		Crude OR (95% CI)	Adjusted OR (95% CI)	Prescription Opioids^c^		Crude OR (95% CI)	Adjusted OR (95% CI)
	Positive (*n* = 3278) No. (%)	Negative (*n* = 44,324) No. (%)			Positive (*n* = 341) No. (%)^d^	Negative (*n* = 7540) No. (%)^d^		
Age (years)								
15–24	322 (9.8)	9975 (22.5)	Reference	Reference	29 (9.7)	2156 (28.9)	Reference	Reference
25–39	1114 (34.0)	13388 (30.2)	2.58 (2.27, 2.93)	2.80 (2.44, 3.20)	118 (38.5)	2424 (33.2)	3.45 (2.02, 5.87)	3.77 (2.23, 6.38)
40–64	1490 (45.5)	16105 (36.3)	2.87 (2.53, 3.24)	3.00 (2.63, 3.42)	154 (43.5)	2363 (32.7)	3.95 (2.45, 6.35)	4.16 (2.66, 6.50)
≥ 65	352 (10.7)	4856 (11.0)	2.25 (1.92, 2.62)	2.12 (1.79, 2.50)	27 (8.3)	406 (5.2)	4.75 (2.24, 10.06)	4.62 (2.19, 9.74)
Sex								
Male	2409 (73.5)	34,569 (78.0)	Reference	Reference	178 (52.0)	4349 (56.8)	Reference	Reference
Female	869 (26.5)	9748 (22.0)	1.28 (1.18, 1.39)	1.25 (1.14, 1.36)	156 (48.0)	3092 (43.2)	1.21 (0.90, 1.64)	1.21 (0.91, 1.60)
Race/ethnicity								
Non-Hispanic White	2766 (92.5)	33,265 (83.2)	Reference	Reference	266 (77.2)	4737 (60.3)	Reference	Reference
Non-Hispanic Black	159 (5.3)	4796 (12.0)	0.40 (0.34, 0.47)	0.36 (0.30, 0.42)	51 (19.0)	1508 (26.1)	0.57 (0.39, 0.83)	0.51 (0.35, 0.75)
Other	65 (2.2)	1935 (4.8)	0.40 (0.32, 0.52)	0.47 (0.36, 0.60)	14 (3.8)	1216 (13.6)	0.22 (0.10, 0.49)	0.24 (0.11, 0.53)
Geographic region								
South	1688 (51.5)	19,166 (43.2)	Reference	Reference	92 (37.7)	1894 (41.3)	Reference	Reference
Midwest	676 (20.6)	9711 (21.9)	1.17 (1.01, 1.34)	0.92 (0.78, 1.09)	91 (25.6)	1816 (20.3)	1.38 (0.87, 2.19)	1.36 (0.87, 2.14)
Northeast	302 (9.2)	5057 (11.4)	1.48 (1.30, 1.67)	1.29 (1.10, 1.51)	73 (21.6)	1379 (17.3)	1.37 (0.83, 2.28)	1.22 (0.73, 2.04)
West	612 (18.7)	10,390 (23.4)	0.99 (0.86, 1.14)	0.81 (0.68, 0.96)	85 (15.1)	2451 (21.1)	0.78 (0.47, 1.30)	1.02 (0.64, 1.63)
Testing positive for marijuana								
No	2751 (83.9)	37,396 (84.4)	Reference	Reference	305 (88.0)	7020 (92.3)	Reference	Reference
Yes	527 (16.1)	6928 (15.6)	1.03 (0.94, 1.14)	1.28 (1.15, 1.42)	36 (12.0)	520 (7.7)	1.62 (1.04, 2.51)	2.03 (1.29, 3.20)
Testing positive for alcohol								
No	2170 (66.2)	25,436 (57.4)	Reference	Reference	326 (97.3)	7134 (94.6)	Reference	Reference
Yes	1108 (33.8)	18,888 (42.6)	0.69 (0.64, 0.74)	0.68 (0.63, 0.74)	15 (2.7)	406 (5.4)	0.50 (0.22, 1.14)	0.56 (0.24, 1.31)

^a^There were 7 drivers with missing data on gender and 4616 on race from the FARS

^b^There were 204 drivers with missing data on age, 106 on gender, and 89 on race from the NRS

^c^Unweighted frequencies of drivers by prescription opioid testing results in the sample

^d^Percentage of drivers weighted for the US driver population

Among the 7881 drivers in the NRS sample, 4.5% tested positive for prescription opioids, 7.9% positive for marijuana, and 5.3% positive for alcohol. The majority of the drivers were male, non-Hispanic white, and under 40 years of age (Table 1). Drivers who tested positive for marijuana were more likely to test positive for prescription opioids (adjusted OR = 2.03, 95% CI = 1.29–3.20) and drivers who tested positive for alcohol were less likely to test positive for prescription opioids (adjusted OR = 0.56, 95% CI = 0.24–1.31). Drivers who were female or non-Hispanic white had significantly elevated risk of testing positive for prescription opioids (Table 2). The odds of testing positive for prescription opioids increased with age (Table 2). Relative to drivers who tested negative for marijuana, those testing positive for marijuana were 66% more likely to test positive for alcohol (adjusted OR = 1.66, 95% CI = 1.07–2.58). In both the FARS and the NRS study samples, the association of marijuana use with increased odds of prescription opioid use existed in different strata of drivers (Supplementary Table S1).

## Discussion

In this study, we attempted to address the marijuana-prescription opioids substitution hypothesis by analyzing toxicological testing data from two US national samples of drivers. Our results revealed that marijuana use is not associated with decreased odds of prescription opioid use among fatally injured drivers and in a nationally representative sample of drivers. On the contrary, marijuana use appears to be associated with increased use of prescription opioids. The results are consistent between the FARS and the NRS samples and held robust across strata of driver characteristics. The concurrent use of marijuana and opioids detected from the two study samples of drivers is suggestive of a supplementary rather than a substitutive relationship between these two substances.

Our study adds more empirical evidence for assessing the marijuana-prescription opioids substitution hypothesis [19, 25–29, 31, 40, 54]. As more state governments and policy makers consider legalizing marijuana as a harm reduction strategy for controlling the opioid epidemic, results from our study underscore the importance for additional studies to validate the assumptions about the relationship between marijuana and prescription opioids. If confirmed by further research, the supplementary relationship between marijuana and prescription opioids implies that legalizing marijuana as a policy intervention to control the opioid epidemic might be misguided and potentially counterproductive.

Our findings are consistent with those from the large cohort study that found no evidence that marijuana use was associated with a reduction in prescription opioid use among 1514 patients with chronic pain [33]. Similarly, our results are also consistent with those from a nationally representative survey of 57,146 participants wherein medical marijuana use was associated with increased use and misuse of prescription opioids [34]. Conversely, results of our study are divergent from those based on self-reported data that support the substitution hypothesis. Specifically, surveys conducted in the United States [19, 25, 29–32] and Canada [26–28] have found high rates of self-reported substitution of marijuana for prescription opioids among recreational and medical marijuana users. In addition, two prospective open-label trials reported an association of medicinal marijuana use and improved pain outcomes among chronic pain patients, suggesting that marijuana may augment the analgesic effects of prescription opioids [54, 55]. Ecological studies based on aggregate data have reported opioid-related benefits (e.g., reduced opioid prescriptions) associated with state marijuana laws [35–39]. Given the widespread acceptance of marijuana use to relieve pain and reduce the risks of addiction and overdose associated with prescription opioids, it is plausible that patients may perceive marijuana as a safer substitute with fewer side effects and limited risk of overdose [56]. It may also be possible for some chronic pain patients to experience relief with marijuana since the cannabinoid and opioid receptor systems are anatomically and biochemically similar and the presence of opioids appear to augment the analgesic effects of marijuana [55].

The conflicting findings in the research literature are due in part to differences in measurement ascertainments and study designs. First, all surveys were based on self-reported data rather than toxicological testing data. As such, self-reported data from survey studies are susceptible to misclassification, recall, or social desirability biases. Second, many survey studies were based on small sample sizes [19, 26, 27, 29, 54], had low response rates [26, 28–30], and/or relied on self-selecting convenience samples [25, 27, 31, 32], leading to non-response bias, selection bias and volunteer bias which may further threaten the validity of the results. Third, patients in survey studies were often recruited through medical marijuana organizations or organizations that support medical marijuana [19, 26–29, 32], which may result in overrepresentation of subjects who are biased towards marijuana use. Lastly, online solicitation for study participants in some survey studies [19, 26, 28, 29, 32] would likely miss those without internet access and those with privacy concerns. Research with rigorous designs, such as longitudinal studies and randomized clinical trials, is needed to guide and inform drug policy.

Our study has several limitations. First, the cross-sectional study design made it impossible to elicit temporality in the use of different drugs. However, prescription opioids have relatively shorter half-lives of 2–4 h compared to marijuana, which can be present for days after the last use. Second, it is possible that concurrent use of marijuana and prescription opioids may have led to fatal motor vehicle crashes, which may introduce collider bias to the results from the FARS sample. However, the inclusion/exclusion criteria were independent of drug testing results and crash responsibility. Third, it is possible that mechanisms other than supplementarity may explain the observed positive association between marijuana use and prescription opioid use. For instance, some drivers might use marijuana to taper off prescription opioids (i.e., reduce the dosage), resulting in positive tests for both substances. Fourth, our study samples are fatally injured drivers and randomly selected drivers, which are different from patients with chronic pain included in previous studies. Finally, our study samples are limited to drivers with toxicological testing results, making our findings potentially susceptible to selection bias. Given that drivers included in the study are largely comparable to those excluded with respect to demographic characteristics and blood alcohol concentrations, selection bias is unlikely to pose a serious threat to the validity of our findings.

## Conclusions

In this study of two US national samples of drivers, marijuana use is not associated with decreased odds of prescription opioid use. On the contrary, marijuana use is associated with significantly increased odds of prescription opioid use as well as alcohol use in drivers. Existent evidence supporting the marijuana-prescription opioids substitution hypothesis is based primarily on ecological studies and survey studies of patients with chronic pain. As more states move toward legalizing marijuana as a harm reduction strategy for controlling the opioid epidemic, there is an urgent need for better understanding the relationship between marijuana use and use of prescription opioids through rigorous study designs and objectively measured longitudinal data from different population groups.

## Supplementary information

**Additional file 1: Table S1.** Estimated adjusted odds ratios (ORs) and 95% confidence intervals (CIs) of prescription opioid positivity according to driver demographic characteristics, 2011–16 Fatality Analysis Reporting System (FARS) and 2013–14 National Roadside Survey of Alcohol and Drug Use by Drivers (NRS).

## Data Availability

The data for this study come from the Fatality Analysis Reporting System and the National Roadside Survey of Alcohol and Drug Use by Drivers. Both data sets are available from the National Highway Traffic Safety Administration (Washington, DC). For information on obtaining this data, visit https://www.nhtsa.gov/research-data/fatality-analysis-reporting-system-fars (FARS) and https://www.nhtsa.gov/2013-14-national-roadside-study-alcohol-and-drug-use-drivers/2013-14-nrs-alcohol-and-drug-use (NRS).

## Abbreviations

BAC: Blood alcohol concentration
CI: Confidence interval
FARS: Fatality Analysis Reporting System
NRS: National Roadside Survey of Alcohol and Drug Use by Drivers
OR: Odds ratio
THC: Delta-9-tetrahydrocannabinol

## References

[1] Bipartisan Policy Center (2019). Tracking Federal Funding to combat the opioid crisis.

[2] Ahmad FB, Escobedo LA, Rossen LM, Spencer MR, Warner M, Sutton P (2019). Provisional drug overdose death counts [internet].

[3] McClure D, Paddock E (2018). Insights from early state efforts to address the opioid crisis.

[4] Office of National Drug Control Policy (2011). Epidemic: Responding to America’s Prescription Drug Abuse Crisis.

[5] Hedegaard H, Warner M, Minino AM (2017). Drug Overdose Deaths in the United States, 1999-2006.

[6] Centers for Disease Control and Prevention (2018). U.S. Opioid Prescribing Rate Maps.

[7] Centers for Disease Control and Prevention (2019). Overdose Death Maps.

[8] Centers for Disease Control and Prevention (2018). Understanding the Epidemic.

[9] Centers for Disease Control and Prevention (2017). Addressing the Prescription Opioid Crisis: CDC Rx Awareness Campaign Overview.

[10] US Department of Health and Human Services, Office of the Surgeon General (2018). Facing Addiction in America: the Surgeon General’s Spotlihgt on Opioids.

[11] Florence C, Luo F, Xu L, Zhou C (2018). The economic burden of prescription opioid overdose, abuse and depedence in the United States, 2013. Med Care.

[12] Voelker R (2018). States move to substitute opioids with medical marijuana to quell epidemic. JAMA.

[13] Fiore MC, Smith SS, Jorenby DE, Baker TB (1994). The effectiveness of the nicotine patch for smoking cessation: a meta-analysis. JAMA.

[14] Mattick RP, Breen C, Kimber J, Davoli M (2009). Methadone maintenance therapy versus no opioid repalcement therapy for opioid dependence. Cochrane Database Syst Rev.

[15] Hurd YL, O'Brien CP (2018). Molecular genetics and new medication strategies for opioid addiction. Am J Psychiatry.

[16] Socias ME, Wood E, Lake S, Nolan S, Fairbairn N, Hayashi K (2018). High-intensity cannabis use is associated with retention in opioid agonist treatment: a longitudinal analysis. Addiction.

[17] New York State Department of Health (2018). New York State Department of Health announces opioid replacement now a qualifying condition for medical marijuana.

[18] National Conference of State Legislatures (2019). State Medical Marijuana Laws.

[19] Boehnke KF, Litinas E, Clauw DJ (2016). Medical cannabis use is associated with decreased opiate medication use in a retrospective cross-sectional survey of patients with chronic pain. J Pain.

[20] Grinspoon P (2018). Access to medical marijuana reduces opioid prescriptions [internet].

[21] Ault A (2019). Endorsing cannabis as an opioid substitute ‘irresponsible’.

[22] Frakt A (2019). Can marijuana help cure the opioid crisis? The New York Times.

[23] Humphreys K, Saitz R (2019). Should physicians recommend replacing opioids with cannabis?. JAMA.

[24] Reinberg S (2019). Pot a substitute to opioids or sleep meds for many. HealthDay News.

[25] Corroon JM, Mischley LK, Sexton M (2017). Cannabis as a substitute for prescription drugs-a cross-sectional study. J Pain Res.

[26] Lucas P, Walsh Z (2017). Medical cannabis access, use, and substitution for prescription opioids and other substances: a survey of authorized medical cannabis patients. Int J Drug Policy.

[27] Lucas P, Walsh Z, Crosby K, Callaway R, Belle-Isle L, Kay R (2016). Substituting cannabis for prescription drugs, alcohol and other substances among medical cannabis patients: the impact of contextual factors. Drug Alcohol Rev.

[28] Lucas P, Baron EP, Jikomes N (2019). Medical cannabis patterns of use and substitution for opioids & other pharmaceutical drugs, alcohol, tobacco, and illicit substances; results from a crosssectional survey of authorized patients. Harm Reduct J.

[29] Reiman A (2007). Medical Cannabis patients: patient profiles and health care utilization patterns. Complement Health Pract Rev.

[30] Reinarman C, Nunberg H, Lanthier F, Heddleston T (2011). Who are medical marijuana patients? Population characteristics from nine California assessment clinics. J Psychoactive Drugs.

[31] Sexton M, Cuttler C, Finnell JS, Mischley LK (2016). A cross-sectional survey of medical cannabis users: patterns of use and perceived efficacy. Cannabis Cannabinoid Res.

[32] Piper BJ, DeKeuster RM, Beals ML, Cobb CM, Burchman CA, Perkinson L (2017). Substitution of medical cannabis for pharmaceutical agents for pain, anxiety, and sleep. J Psychopharmacol.

[33] Campbell G, Hall WD, Peacok A, Lintzeris N, Bruno R, Larance B (2018). Effect of cannabis use in people with chronic non-cancer pain prescribed opioids: findings from a 4-year prospective cohort study. Lancet Public Health.

[34] Caputi Theodore L., Humphreys Keith (2018). Medical Marijuana Users are More Likely to Use Prescription Drugs Medically and Nonmedically. Journal of Addiction Medicine.

[35] Bradford AC, Bradford WD (2017). Medical marijuana Laws may be associated with a decline in the number of prescriptions for Medicaid enrollees. Health Aff (Millwood).

[36] Bradford AC, Bradford WD, Abraham A, Bagwell AG (2018). Association between US state medical Cannabis Laws and Opioid prescribing in the Medicare part D population. JAMA Intern Med.

[37] Shi Y (2017). Medical marijuana policies and hospitalizations related to marijuana and opioid pain reliever. Drug Alcohol Depend.

[38] Bachhuber MA, Saloner B, Cunningham CO, Barry CL (2014). Medical cannabis laws and opioid analgesic overdose mortality in the United States, 1999-2010. JAMA Intern Med.

[39] Powell D, Pacula RL, Jacobson M (2018). Do medical marijuana laws reduce addictions and deaths related to pain killers?. J Health Econ.

[40] Chihuri S, Li G (2019). State marijuana laws and opioid overdose mortality. Inj Epidemiol.

[41] Caputi TL, Sabet KA (2018). Population-level analyses cannot tell us anything about individual-level marijuana-opioid. substitution. Am J Public Health.

[42] National Highway Traffic Safety Administration (2016). Fatality analysis reporting system analytical user’s manual 1975-2015.

[43] National Highway Traffic Safety Administration (2018). 2017 FARS/CRSS coding and validation manual.

[44] Lacey JH, Kelley-Baker T, Furr-Holden D, Voas R, Moore C, Brainard K (2009). 2007 national roadside survey of alcohol and drug use by drivers: methodology.

[45] Kelley-Baker T, Lacey JH, Berning A, Ramirez A, Moore C, Brainard K (2016). 2013–2014 National Roadside Study of alcohol and drug use by drivers: methodology.

[46] Kaplan J, Kraner J, Paulozzi L (2006). Alcohol and other drug use among victims of motor-vehicle crashes-West Virginia, 2004–2005. MMWR Morb Mortal Wkly Rep.

[47] Ruiz L, Posey BM, Neuilly MA, Stohr MK, Hemmens C (2018). Certifying death in the United States. J Forensic Sci.

[48] Davis G, Hanzlick R, Denton J (2015). The medical examiner and coroner systems.

[49] Li G, Brady JE, Chen Q (2013). Drug use and fatal motor vehicle crashes: a case-control study. Accid Anal Prev.

[50] Drug Enforcement Administration (2019). Controlled substance schedules.

[51] Jin H, Williams SZ, Chihuri S, Li G, Chen Q (2018). Validity of oral fluid test for Delta-9-tetrahydrocannabinol in drivers using the 2013 National Roadside Survey Data. Inj Epidemiol.

[52] Moore C, Vincent M, Rana S, Coulter C, Agrawal A, Soares J (2006). Stability of Delta (9)-tetrahydrocannabinol (THC) in oral fluid using the Quantisal collection device. Forensic Sci Int.

[53] Moore C, Rana S, Coulter C (2007). Determination of meperidine, tramadol and oxycodone in human oral fluid using solid phase extraction and gas chromatography-mass spectrometry. J Chromatogr B Anal Technol Biomed Life Sci.

[54] Haroutounian S, Ratz Y, Ginosar Y, Furmanov K, Saifi F, Meidan R, Davidson E (2016). The effect of medicinal cannabis on pain and quality-of-life outcomes in chronic pain: a prospective open-label study. Clin J Pain.

[55] Abrams DI, Couey P, Shade SB, Kelly ME, Benowitz NL (2011). Cannabinoid-opiod interaction in chronic pain. Clin Pharmacol Ther.

[56] Zaller N, Topletz A, Frater S, Yates G, Laly M (2015). Profiles of medicinal cannabis patients attending compassion centers in Rhode Island. J Pyschoactive Drugs.

