# Supplementary material for: Is marijuana use associated with decreased use of prescription opioids? Toxicological findings from two US national samples of drivers
Source: Subst Abuse Treat Prev Policy. 2020 Feb 17;15:12. doi: 10.1186/s13011-020-00257-7 (PMC7027272; doi:10.1186/s13011-020-00257-7)
Supplement: Supplementary file 1 — Additional file 1: Table S1. Estimated adjusted odds ratios (ORs) and 95% confidence intervals (CIs) of prescription opioid positivity according to driver demographic characteristics, 2011–16 Fatality Analysis Reporting System (FARS) and 2013–14 National Roadside Survey of Alcohol and Drug Use by Drivers (NRS). [file 13011_2020_257_MOESM1_ESM.docx]

**Table S1**. Estimated adjusted odds ratios (ORs) and 95% confidence intervals (CIs) of prescription opioid positivity according to driver demographic characteristics, 2011-16 Fatality Analysis Reporting System (FARS) and 2013-14 National Roadside Survey of Alcohol and Drug Use by Drivers (NRS).

| Characteristic | FARS^a^ | NRS^b^ |
| --- | --- | --- |
|  | Estimated OR^c^  (95% CI) | Estimated OR^c^  (95% CI) |
|  |  |  |
| Age (years) |  |  |
| 15-24 | 2.05 (1.55, 2.70) | 3.84 (1.42, 10.43) |
| 25-39 | 1.04 (0.87, 1.23) | 2.31 (1.22, 4.37) |
| 40-64 | 1.28 (1.08, 1.52) | 0.94 (0.45, 1.97) |
| ≥65 | 2.25 (1.27, 3.98) | n/a |
| Sex |  |  |
| Male | 1.32 (1.17, 1.48) | 2.09 (1.31, 3.33) |
| Female | 1.05 (0.81, 1.35) | 1.82 (0.73, 4.54) |
| Race/ethnicity |  |  |
| Non-Hispanic White | 1.26 (1.13, 1.42) | 1.51 (0.90, 2.54) |
| Non-Hispanic Black | 1.53 (1.03, 2.28) | 2.19 (0.80, 6.01) |
| Other | 1.24 (0.58, 2.64) | 16.25 (3.13, 84.29) |
| Geographic region |  |  |
| Northeast | 1.15 (0.77, 1.72) | 2.16 (0.80, 5.81) |
| Midwest | 1.30 (1.04, 1.62) | 1.85 (0.83, 4.12) |
| South | 1.27 (1.09, 1.49) | 2.55 (1.30, 5.00) |
| West | 1.19 (0.94, 1.52) | 0.91 (0.26, 3.21) |

^a^There were 7 drivers with missing data on gender and 4616 on race from the FARS.

^b^There were 204 drivers with missing data on age, 106 on gender, and 89 on race from the NRS.

^c^Drivers who tested negative for marijuana were used as the reference group.
